# Distribution of ossified spinal lesions in patients with severe ossification of the posterior longitudinal ligament and prediction of ossification at each segment based on the cervical OP index classification: a multicenter study (JOSL CT study)

**DOI:** 10.1186/s12891-018-2009-7

**Published:** 2018-04-05

**Authors:** Takashi Hirai, Toshitaka Yoshii, Narihito Nagoshi, Kazuhiro Takeuchi, Kanji Mori, Shuta Ushio, Akio Iwanami, Tsuyoshi Yamada, Shoji Seki, Takashi Tsuji, Kanehiro Fujiyoshi, Mitsuru Furukawa, Soraya Nishimura, Kanichiro Wada, Takeo Furuya, Yukihiro Matsuyama, Tomohiko Hasegawa, Katsushi Takeshita, Atsushi Kimura, Masahiko Abematsu, Hirotaka Haro, Tetsuro Ohba, Masahiko Watanabe, Hiroyuki Katoh, Kei Watanabe, Hiroshi Ozawa, Haruo Kanno, Shiro Imagama, Kei Ando, Shunsuke Fujibayashi, Masao Koda, Masashi Yamazaki, Morio Matsumoto, Masaya Nakamura, Atsushi Okawa, Yoshiharu Kawaguchi

**Affiliations:** 1 Department of Orthopedic Surgery, Tokyo Medical and Dental University, 1-5-45 Yushima, Bunkyo-ku, Tokyo, 113-8519 Japan; 2 Department of Orthopedic Surgery, School of Medicine, Keio University, 35 Shinanomachi, Shinjuku-ku, Tokyo, 160-8582 Japan; 3 Department of Orthopedic Surgery, National Hospital Organization Okayama Medical Center, 1711-1 Tamasu, Okayama, Okayama 701-1154 Japan; 4 Department of Orthopedic Surgery, Shiga University of Medical Science, Tsukinowa-cho, Seta, Otsu, Shiga 520-2192 Japan; 5 Department of Orthopedic Surgery, Faculty of Medicine, University of Toyama, 2630 Sugitani, Toyama, Toyama 930-0194 Japan; 6 Department of Orthopedic Surgery, Fujita Health University, 1-98 Dengakugakubo, Kutsukake, Toyoake, Aichi 470-1192 Japan; 7 Department of Orthopedic Surgery, National Hospital Organization Murayama Medical Center, 2-37-1 Gakuen, Musashimurayama, Tokyo, 208-0011 Japan; 8 Department of Orthopedic Surgery, Shizuoka City Shimizu Hospital, 1231 Miyakami, Shimizu-ku, Shizuoka, 424-8636 Japan; 9 Department of Orthopedic Surgery, Keiyu Hospital, 3-7-3 Minatomirai, Nishi-ku, Yokohama, Kanagawa 220-0012 Japan; 10 Department of Orthopedic Surgery, Hirosaki University Graduate School of Medicine, 53 Honcho, Hirosaki, Aomori 036-8203 Japan; 11 Department of Orthopedic Surgery, Chiba University Graduate School of Medicine, 1-8-1 Inohana, Chuo-ku, Chiba, Chiba 260-0856 Japan; 12 Department of Orthopedic Surgery, Hamamatsu University School of Medicine, 1-20-1 Handayama, Hamamatsu, Shizuoka 431-3125 Japan; 13 Department of Orthopedics, Jichi Medical University, 3311-1 Yakushiji, Shimotsuke, Tochugi 329-0498 Japan; 14 Department of Orthopedic Surgery, Graduate School of Medicine and Dental Science, Kagoshima University, 8-35-1 Sakuragaoka, Kagoshima, Kagoshima 890-8520 Japan; 15 Department of Orthopedic Surgery, University of Yamanashi, 1110 Shimokato, Chuo-ku, Yamanashi, 409-3898 Japan; 16 Department of Orthopedic Surgery, Surgical Science, Tokai University School of Medicine, 143 Shimokasuya, Isehara, Kanagawa 259-1143 Japan; 17 Department of Orthopedic Surgery, Niigata University Medicine and Dental General Hospital, 1-754 Asahimachidori, Chuo-ku, Niigata, Niigata 951-8520 Japan; 18 Department of Orthopedic Surgery, Tohoku Medical and Pharmaceutical University, 4-4-1 Komatsushima, Aobaku, Sendai, Miyagi 981-8558 Japan; 19 Department of Orthopaedic Surgery, Tohoku University School of Medicine, 1-1 Seiryomachi, Aoba-ku, Sendai, Miyagi 980-8574 Japan; 20 Department of Orthopedic Surgery, Nagoya University Graduate School of Medicine, 65 Tsurumaicho, Showa-ku, Nagoya, Aichi 466-0065 Japan; 21 Department of Orthopedic Surgery, Graduate School of Medicine, Kyoto University, 54 Kawaharacho, Shogoin, Sakyo-ku, Kyoto, Kyoto 606-8507 Japan; 22 Department of Orthopedic Surgery, Faculty of Medicine, University of Tsukuba, 2-1-1 Amakubo, Tsukuba, Ibaraki 305-8576 Japan; 23 Japanese Organization of the Study for Ossification of Spinal Ligament (JOSL), 1-5-45 Yushima, Bunkyo-ku, Tokyo, 113-8519 Japan

**Keywords:** OPLL, Computed tomography, Whole spine, Ossification predisposition, Prevalence

## Abstract

**Background:**

In patients with ossification of the posterior longitudinal ligament (OPLL) in the cervical spine, it is well known that the thoracic ossified lesions often coexist with the cervical lesions and can cause severe myelopathy. However, the prevalence of OPLL at each level of the thoracic and lumbar spinal segments is unknown. The aims of this study were to investigate how often OPLL occurs at each level in the thoracolumbar spine in patients with a radiological diagnosis of cervical OPLL and to identify the spinal levels most likely to develop ossification.

**Methods:**

Data were collected from 20 institutions in Japan. Three hundred and twenty-two patients with a diagnosis of cervical OPLL were included. The OPLL index (OP index), defined as the sum of the vertebral body and intervertebral disc levels where OPLL is present, was used to determine disease severity. An OP index ≥20 was defined as severe OPLL. The prevalence of OPLL at each level of the thoracic and lumbar spinal segments was calculated.

**Results:**

Women were more likely to have ossified lesions in the thoracolumbar spine than men. Severe OPLL was significantly more common in women than in men (20% vs. 4.5%). For thoracic vertebral OPLL, the most frequently affected was the T1 segment in both men and women, followed by the T1/2 and T3/4 intervertebral levels in men and women, respectively. Ossified lesions were frequently seen at the intervertebral and vertebral levels around the cervicothoracic and thoracolumbar junctions in men with severe OPLL, whereas OPLL was more diffusely distributed in the thoracic spine in women with severe OPLL.

**Conclusion:**

Thoracolumbar OPLL occurred most often at T1 in men and at T3/4 in women. In severe OPLL cases, although ossified lesions were frequently seen at the intervertebral and vertebral levels around the cervicothoracic and thoracolumbar junctions in men, OPLL could be observed more diffusely in the thoracic spine in women.

## Background

Ossification of the spinal ligaments is a form of heterotopic ossification that occurs throughout the spine. Ossification of the posterior longitudinal ligament (OPLL) involves the spinal canal and often leads to myelopathy [1, 2]. Cervical OPLL, which can be detected easily on plain radiographs, has been investigated in detail as far back as the nineteenth century [3]. Ossified lesions in the thoracic spine sometimes coexist with cervical OPLL and cause severe symptoms. However, it is difficult to diagnose pathologic conditions of the thoracic spine on radiographs alone, so the prevalence of thoracic OPLL remains unclear [4]. Recently, multidetector computed tomography (CT) has been found to be the most suitable modality for identifying ossification in the thoracic spine, regardless of any superimposed thoracic complexities. Nevertheless, there is no detailed multicenter research on the prevalence of thoracolumbar OPLL. The purpose of this study was to investigate the prevalence of ossified lesions in the thoracolumbar spine in patients with cervical OPLL at multiple institutions. We also investigated how frequently OPLL occurred at each spinal level and attempted to clarify which levels of the spine are more predisposed to OPLL lesions.

## Methods

### Patients and methods

**Fig. 1:**
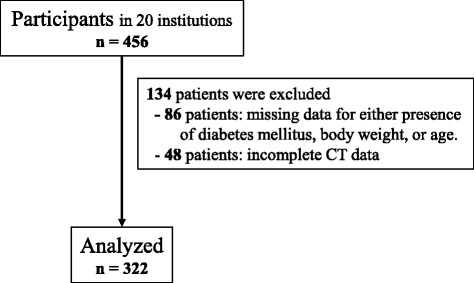
Patient flow diagram

The study included patients with a radiographic diagnosis of OPLL involving the cervical spine as well as symptoms, such as neck pain, numbness in the upper and/or lower extremities, and clumsiness or gait disturbance. Each patient underwent whole-spine CT imaging at any one of the 20 institutions where members of the research team worked. Patients who had undergone anterior decompression surgery for OPLL and patients younger than 15 years of age were excluded. Four hundred and fifty-six patients with OPLL were identified, of whom only 322 had full demographic and anthropometric data available for analysis (Fig. 1). The study involved 242 men and 80 women with a mean age of 64.6 (range 30–93) years. The study was approved by the institutional review board at each institution. Informed consent was obtained from all patients before enrollment in the study.

### Evaluations

Basic clinical data were collected for all patients, including for age, sex, presence of diabetes, and body mass index (BMI). CT images of the cervical, thoracic, and lumbosacral spine, from the occipital bone to the sacrum, were obtained in each patient. The prevalence of OPLL in the cervical spine from the clivus to C7 and in other spinal regions from T1 to S1 was evaluated on mid-sagittal CT images. The analysis was independently performed by five senior spine surgeons (TH, KT, KM, AI, TY). Prior to reviewing the images for this study, all of the readers interpreted the same images for 20 patients to check interobserver agreement. The average kappa coefficient was 0.76 (0.71–0.81), which indicated good agreement and was consistent with the findings of a previous study [5]. Ossified lesions were recorded at each vertebral body and at the intervertebral disc level. An OPLL index (OP index) [5], defined as the sum of the vertebral body and intervertebral disc levels where OPLL is present, was calculated according to the method described in a previous report [5]. Patients with an OP index ≥20 were deemed to have severe OPLL. We also defined the sum of the levels at which OPLL was present in the cervical spine as the cervical OP index. Using a previously reported method [6], we divided patients into three groups according to their cervical OP index values, namely, grade 1 (≤5), grade 2 (6–9), and grade 3 (≥10). The physical and radiologic data were compared between the male and female patient populations. Furthermore, we investigated whether there was an association between the cervical OP index grade and the prevalence of thoracolumbar OPLL at each level.

### Statistical analysis

Student’s unpaired *t* test was used to identify statistically significant differences in age, BMI, and the OP index for the cervical spine between men and women. Chi-squared test was used to test for a sex-related difference in the presence of diabetes. Tukey’s post hoc test was applied to compare the three groups classified according to cervical OP grade. A forward stepwise logistic regression model was used to investigate whether the cervical OP grade could predict the presence of OPLL in the thoracolumbar spine and the prevalence of patients with an OP index of ≥20 [7]. All statistical analyses were performed using SPSS for Windows version 22.0 (IBM Corp., Armonk, NY). All data are expressed as the mean ± standard deviation. A *p*-value < 0.05 was considered to be statistically significant.

## Results

### Demographic data

Patient demographic characteristics are summarized in Table 1. Mean patient age was 64.7 years. Mean BMI was 25.7 and 31.7% (102/233 patients) of the patients had diabetes mellitus. Using the CT classification, the cervical OP index was grade 1 in 169 patients (52.5%), grade 2 in 107 patients (33.2%), and grade 3 in 46 patients (14.3%). The mean cervical and whole-spine OP index values were 5.83 and 9.21, respectively. Twenty-seven patients (8.3%) had an OP index ≥20. Severe OPLL was significantly more common in women than in men (16 patients, 20% vs. 11 patients, 4.5%, *p* < 0.01). There was no significant sex-related difference in age, prevalence of diabetes mellitus, or BMI. Interestingly, the OP index for the whole spine was significantly higher in women than in men (9.2 vs. 8.2, *p* < 0.01). However, there was no statistically significant sex-related difference in the cervical OP index.

**Table 1:** Demographics of male and female patients

	Male + Female (*n* = 322)	Male (*n* = 242)	Female (*n* = 80)	P (M vs F)
Age (years old)	64.7 ± 11.2	64.7 ± 11.6	64.6 ± 10.0	0.90
Diabetes Mellitus (%)	31.7%	31.8%	31.3%	0.92
BMI	25.7 ± 4.8	25.8 ± 4.8	25.5 ± 4.7	0.62
JOSL CT classification				
Grade 1 (1 < cervical OP-index≤5)	169 (52.5%)	125 (51.7%)	44 (55%)	–
Grade 2 (6 < cervical OP-index ≤9)	107 (33.2%)	83 (34.3%)	24 (30%)	–
Grade 3 (10 < cervical OP-index)	46 (14.3%)	34 (14.0%)	12 (15%)	–
Cervical OP-index	5.83 ± 2.9	5.86 ± 2.9	5.75 ± 3.0	0.78
OP-index	9.21 ± 6.8	8.24 ± 5.5	12.1 ± 9.0	< 0.01
No. of patients with OP-index of > = 20 (%)	27 (8.3%)	11 (4.5%)	16 (20%)	< 0.01

Data are expressed as the mean ± standard deviation; *BMI* body mass index, *OP-index* ossification index of OPLL

### Pattern of distribution of ossified lesions throughout the spine in patients with severe OPLL

The prevalence of OPLL at each level of the spine is shown in Table 2. Thoracic OPLL occurred most often at T1 (14.9%) and at T1/2 (13.6%) in men and at T1 (27.5%) and T3/4 (33.8%) in women. Lumbar OPLL was most common at L1 (5.8%) and T12/L1 (14.5%) in men and at L1 and L2 (both 11.3%) and T12/L1 (27.5%) in women.

**Table 2:**
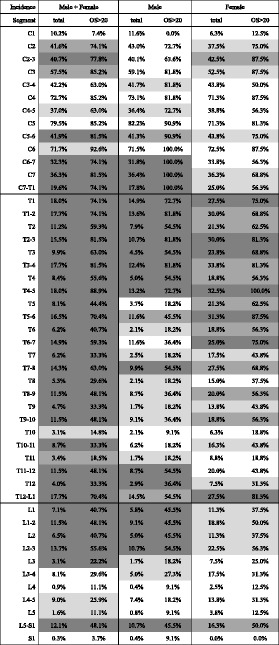
Incidence of ossified lesion in each level

low more significant different (0.01 < *p* < 0.05)

moderate more significant different (0.001 < *p* < 0.01)

high more significant different (*p* < 0.001)

**Fig. 2:**
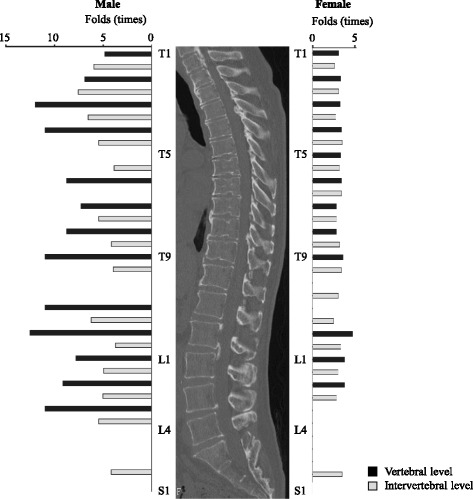
Hazard ratios for the prevalence of ossified lesions at each spinal level in patients with severe OPLL compared with patients with OPLL of any grade. OPLL, ossification of the posterior longitudinal ligament

We then compared the prevalence of OPLL at each level in all patients and that in patients with an OP index ≥20. Overall, the patients with severe OPLL were more likely to have OPLL at the level of the upper cervical spine and at levels from the lower cervical spine to the upper lumbar spine. In men with severe OPLL, ossified lesions were frequently seen at the intervertebral and vertebral levels from C6/7 to T4/5 and from T11/12 to L3/4. However, severe OPLL appeared to be distributed more diffusely in the thoracic spine in women. There were no statistically significant differences in the prevalence of thoracic OPLL at T5, T6/7, T10, and T10/11 in men or at T8, T10, T11, and T11/12 in women. Interestingly, the only significant difference in prevalence of OPLL in the area from the lower lumbar spine to the sacrum was at L5/S1 in both sexes. We also investigated the fold difference in prevalence of ossified lesions at each spinal segment (Fig. 2, Table 3). The prevalence of severe OPLL in the thoracic spine was 3.9–12.6 fold higher in men and 2.3–4.2 fold higher in women. Of note, in men with OPLL affecting the upper thoracic spine, there was a 12.0 fold increase in likelihood at T3 and a 7.6 fold increase at T2/3, and in those with lower thoracic OPLL, there was a 12.6 fold increased likelihood at T12 and a 6.3fold increase at T11/12. Interestingly, although thoracic OPLL had a bimodal distribution in men, the distribution was uniform in women. The distribution pattern in the lumbar spine was very similar between the sexes. However, the prevalence of ossified lesions increased to a greater extent in men (by 4.25–11.0 fold) than in women (by 2.5–3.3 fold). In the lumbosacral region, there was a significantly increased prevalence of ossified lesions only at L5/S1 in both men (4.2 fold) and women (3.1 fold).

**Table 3:** Hazard ratio of the incidence of ossified lesion at each level in patients with OP-index ≧20 compared to total patients

	Male		Female	
Segment	Folds change	p	Folds change	p
T1	4.9	0	2.7	0.0008
T1–2	6.0	0	2.3	0.0079
T2	6.9	0	2.9	0.0023
T2–3	7.6	0	2.7	0.0004
T3	12.0	0	2.9	0.0012
T3–4	6.6	0	2.4	0.0012
T4	11.0	0	3.0	0.0044
T4–5	5.5	0	3.1	0
T5	–	0.1224	2.9	0.0023
T5–6	3.9	0.005	2.8	0.0001
T6	8.8	0.0246	3.0	0.0044
T6–7	–	0.0505	3.0	0.0003
T7	7.3	0.0424	2.5	0.0469
T7–8	5.5	0.0001	2.5	0.0039
T8	8.8	0.0246	–	0.0794
T8–9	4.2	0.0127	2.8	0.0068
T9	11.0	0.0121	3.2	0.0141
T9–10	4.0	0.0161	3.0	0.0044
T10	–	0.628	–	0.2477
T10–11	–	0.3488	2.7	0.0327
T11	11.0	0.0121	–	0.455
T11–12	6.3	0	–	0.0871
T12	12.6	0	4.2	0.0219
T12-L1	3.8	0.0019	3.0	0.0001
L1	7.9	0	3.3	0.0237
L1–2	5.0	0	2.7	0.0186
L2	9.2	0	3.3	0.0237
L2–3	5.1	0.0001	2.5	0.0148
L3	11.0	0.0121	–	0.1003
L3–4	5.5	0.0159	–	0.3594
L4	–	0.1505	–	0.2534
L4–5	–	0.4713	–	0.1779
L5	–	0.2926	–	0.4113
L5-S1	4.2	0	3.1	0.0081
S1	–	0.1505	–	–

### Prevalence of OPLL at each level according to cervical OP index grade

The cervical OP index classification was originally designed to categorize cervical OPLL into three grades of severity according to the sum of all ossifications in the cervical spine. The prevalence of OPLL was investigated at each vertebral and intervertebral segment according to grade to evaluate the usefulness of this classification for prediction of the prevalence of OPLL at each segment in the thoracic and lumbar spines (Table 4). Overall, the prevalence of ossified lesions in the upper thoracic spine and at the thoracolumbar junction increased significantly with increasing grade of severity. The distribution of prevalence of OPLL was similar in men and women. A weak or no association between the prevalence of OPLL and the cervical OP index classification was found in the middle thoracic spine in men. However, statistically significant differences were observed at T4/5, T5/6, T6, T6/7, T8/9, and T9/10 in women. A significant correlation was found at the thoracolumbar junction, except at L2 in men and at T11, L1, L1/2, and L2 in women.

**Table 4:**
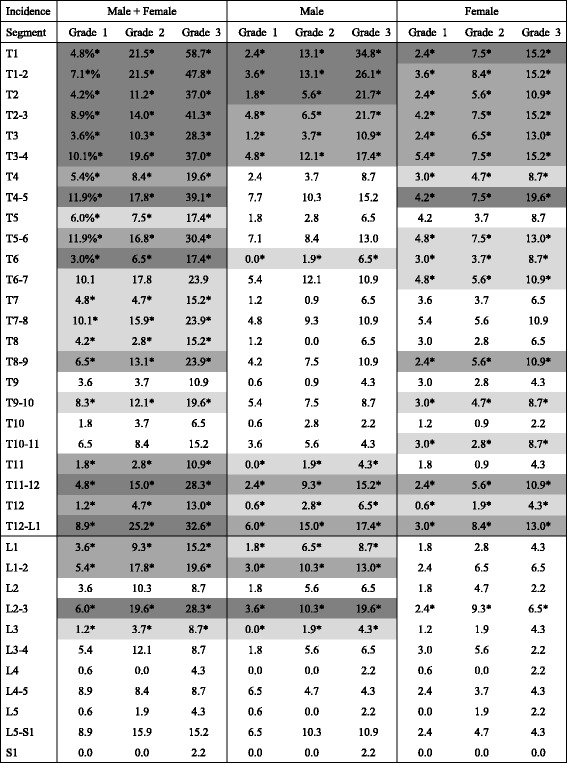
Incidence of ossified lesion in each level according to cervical OP-index grading system

low more significant different (0.01 < *p* < 0.05)

moderate more significant different (0.001 < *p* < 0.01)

high more significant different (*p* < 0.001)

### JOSL-CT grading system for prediction of prevalence of OPLL at each thoracolumbar spinal level

**Fig. 3:**
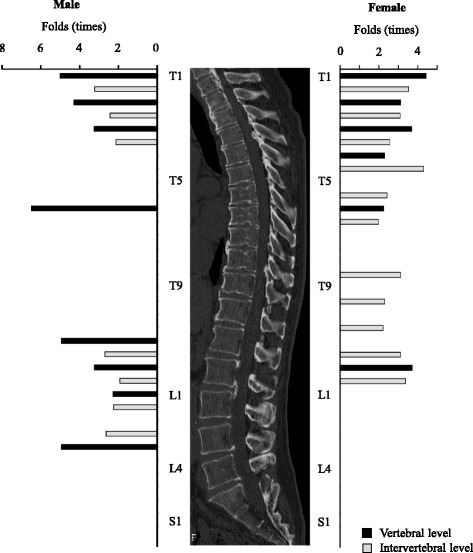
Logistic regression model showing a significant correlation between cervical OP index grade and prevalence of ossification of the posterior longitudinal ligament at different thoracic and lumbar spinal levels

We further investigated the hazard ratios for the prevalence of OPLL at each spinal level where there was a significant correlation between the prevalence of OPLL and the cervical OP index grade (Fig. 3, Table 5). The hazard ratio was 2.1–5.0 in the upper thoracic spine, 6.5 at T6 in the middle thoracic spine in men, and 2.0–4.3 from T4/5 to T6/7 in women. At the thoracolumbar junction in men, the hazard ratio was 2.0–5.0 at both the intervertebral and vertebral segments, but not at the L2 vertebral level. However, a significant correlation (2.1–3.7-fold) was found at T10/11, T11/12, T12, T12/L1, and L2/3 in women.

**Table 5:** Increased risk of OPLL in each level according to cervical OP-index grading system

Grade 1+	Male		Female	
Segment	Odds ratio	p	Odds ratio	p
T1	5.0	0	4.5	0
T1–2	3.2	0	3.5	0.001
T2	4.3	0	3.1	0.003
T2–3	2.5	0.001	3.1	0.002
T3	3.3	0.005	3.7	0.001
T3–4	2.1	0.004	2.6	0.006
T4	–	0.07	2.3	0.027
T4–5	–	0.133	4.3	0
T5	–	0.12	–	0.111
T5–6	–	0.24	2.4	0.01
T6	6.5	0.013	2.3	0.034
T6–7	–	0.084	2.0	0.045
T7	–	0.077	–	0.169
T7–8	–	0.089	–	0.075
T8	–	0.09	–	0.123
T8–9	–	0.078	3.1	0.003
T9	–	0.104	–	0.312
T9–10	–	0.359	2.3	0.027
T10	–	0.25	–	0.234
T10–11	–	0.628	2.2	0.043
T11	5.0	0.036	–	0.153
T11–12	2.7	0.001	3.1	0.003
T12	3.3	0.023	3.7	0.024
T12-L1	2.0	0.007	3.4	0.001
L1	2.3	0.021	–	0.101
L1–2	2.3	0.006	–	0.08
L2	–	0.07	–	0.465
L2–3	2.7	0	2.1	0.041
L3	5.0	0.036	–	0.189
L3–4	–	0.07	–	0.763
L4	–	0.993	–	0.46
L4–5	–	0.449	–	0.381
L5	–	0.47	–	0.118
L5-S1	–	0.24	–	0.312
S1	5.0	0.993	–	–

## Discussion

Various studies [5, 8–10] have reported high concomitance rate in spinal ligament ossification. In this study, we identified the prevalence of OPLL in the thoracic and lumbar spines in patients with cervical OPLL. Kawaguchi et al. [5] demonstrated that more than 50% of patients with cervical OPLL also had OPLL at the thoracic and/or lumbar spine. The prevalence of thoracic OPLL in the general population is reported to be 1.6–1.9% in Japan [4, 8]. This finding suggests that patients with cervical OPLL have a predisposition to hyperostosis in the posterior longitudinal ligament throughout the spine. Therefore, when an ossified lesion is detected in the cervical spine, a whole-spine CT study should be performed to detect lesions in other spinal segments.

Several studies [11–13] have demonstrated that the most frequently involved thoracic site on plain radiography is T6. However, Fujimori et al. [8] reviewed whole-spine CT data for 1500 patients who underwent positron emission tomography and CT (PET-CT) and concluded that the most frequently affected level was T1/2 in men and T5/6 in women. Mori et al. [4] reviewed ossified lesions in patients undergoing chest CT and found that thoracic OPLL was identified most often at T3/4. Our study also demonstrated that thoracic OPLL was most common at T1/2 in men and at T3/4 in women. The likely explanation for the discrepancy between the results of conventional radiographs and those of CT images is that information about the upper thoracic spine is often masked by the superposed bony structures such as the shoulders and ribs. Furthermore, it is known that the posterior longitudinal ligament is thickest and widest in the transitional portion of the cervicothoracic junction [14], so ossification may occur in the upper thoracic spine.

We found that approximately 70–100% of ossified lesions in men with severe OPLL occurred at the cervicothoracic junction, whereas approximately 70–100% of ossified lesions in women with severe OPLL occurred in the middle thoracic spine and approximately 55–75% occurred at the cervicothoracic and thoracolumbar junctions. Furthermore, in women with severe OPLL, ossified lesions tended to occur consistently from the upper thoracic spine to the lumbar spine at a rate approximately 2–3 times that in women with OPLL of any grade. However, the prevalence of ossified lesions in men with severe OPLL was relatively higher at the T3 and T8 vertebral levels and at the thoracolumbar junction (Fig. 2, Table 3). These findings indicate that the presence of ossified lesions at the T3, T8 and thoracolumbar junction might be rare in men (Table 2).

In this study, we categorized patients with cervical OPLL into three groups according to the number of segments at which cervical OPLL could be confirmed. In a previous study [6], we demonstrated that this classification could predict not only the presence but also the degree of hyperostosis in the whole spine. Therefore, we investigated whether there was a correlation between this classification system and the prevalence of ossified lesions at each segment in the thoracolumbar spine. The prevalence of OPLL in the thoracic and lumbar spines increases with increasing severity of cervical hyperostosis. Although OPLL in the upper thoracic spine is generally able to be detected on CT sagittal images, which are often reconstructed, ossification in the thoracolumbar junction cannot be found when patients with cervical OPLL are examined. Park et al. [9] reported coexistence of cervical and thoracic OPLL (tandem calcification) in 33.8% of patients with cervical OPLL, 8.9% of whom subsequently underwent surgery for deterioration of thoracic OPLL. Thoracic stenosis attributable to OPLL is often not recognized or misdiagnosed as lumbar canal stenosis, especially in patients with myelopathic symptoms mainly involving the lower extremities [15]. These reports suggest that patients with a high cervical OP index value are at increased risk of ossified lesions that can cause a spinal disorder. Therefore, whole-spine CT screening is recommended for a patient with cervical OPLL who presents with severe gait disturbance.

Cervical OPLL has occasionally been reported to coexist with not only thoracic OPLL but also ossification of the ligamentum flavum (OLF) [16, 17] and diffuse idiopathic skeletal hyperostosis (DISH) [18, 19]. Fujimori et al. investigated the concomitance of spinal ligament ossification in patients undergoing PET-CT and concluded that the frequencies in patients with cervical OPLL were 13, 34, and 36% for thoracic OPLL, OLF, and DISH, respectively. Mori et al. also reported prevalence of 36 and 8.7% for OLF and DISH in more than 3000 patients undergoing chest CT for investigation of pulmonary disease. There have also been some reports on the prevalence of ossification of other spinal ligaments in patients with cervical OPLL [5, 6]. Kawaguchi et al. reviewed the CT data for 178 patients with cervical OPLL and reported that 64.6% had OLF, while Nishimura et al. evaluated whole-spine CT images for 234 patients with cervical OPLL and found that the prevalence rate of DISH was 48.7%. Thus, ossification of each ligament may influence hyperostosis of each ligament.

This study does have some limitations in that it is based on CT examination of patients with cervical OPLL rather than being a population-based study. Further, we could not evaluate quantitative measurements of clinical symptoms. In addition, we did not determine the distribution of ossification in spinal ligaments other than the OPLL. However, in spite of these limitations, we believe that this study provides important epidemiological information for not only patients with cervical OPLL but also radiologists and spine surgeons.

## Conclusion

This multi-institutional study represents the largest review of whole-spine CT images in patients with cervical OPLL. The most frequent segment was T1 in both men and women for thoracic vertebral OPLL, and T1/2 and T3/4 intervertebral levels in men and women, respectively. Ossified lesions were frequently seen at the intervertebral and vertebral levels around the cervicothoracic and thoracolumbar junctions in men with severe OPLL, but could be observed more diffusely in the thoracic spine in their female counterparts.

## Abbreviations

BMI: Body mass index
CT: Computed tomography
DISH: Diffuse idiopathic skeletal hyperostosis
JOSL: Japanese Organization of the study for ossification of spinal ligament
OLF: Ossification of ligamentum flavum
OPLL: Ossification of posterior longitudinal ligament
PET-CT: Positron emission tomography-computed tomography

## References

[1] Sakai K, Okawa A, Takahashi M, Arai Y, Kawabata S, Enomoto M, Kato T, Hirai T, Shinomiya K (2012). Five-year follow-up evaluation of surgical treatment for cervical myelopathy caused by ossification of the posterior longitudinal ligament: a prospective comparative study of anterior decompression and fusion with floating method versus laminoplasty. Spine.

[2] Matsumoto M, Toyama Y, Chikuda H, Takeshita K, Kato T, Shindo S, Abumi K, Takahata M, Nohara Y, Taneichi H, Tomita K, Kawahara N, Imagama S, Matsuyama Y, Yamazaki M, Okawa A (2011). Outcomes of fusion surgery for ossification of the posterior longitudinal ligament of the thoracic spine: a multicenter retrospective survey: clinical article. J Neurosurg Spine.

[3] Key GA (1838). On paraplegia depending on the ligament of the spine. Guy Hosp Rep.

[4] Mori K, Imai S, Kasahara T, Nishizawa K, Mimura T, Matsusue Y (2014). Prevalence, distribution, and morphology of thoracic ossification of the posterior longitudinal ligament in Japanese: results of CT-based cross-sectional study. Spine.

[5] Kawaguchi Y, Nakano M, Yasuda T, Seki S, Hori T, Kimura T (2013). Ossification of the posterior longitudinal ligament in not only the cervical spine, but also other spinal regions: analysis using multidetector computed tomography of the whole spine. Spine.

[6] Hirai T, Yoshii T, Iwanami A, Takeuchi K, Mori K, Yamada T, Wada K, Koda M, Matsuyama Y, Takeshita K, Abematsu M, Haro H, Watanabe M, Watanabe K, Ozawa H, Kanno H, Imagama S, Fujibayashi S, Yamazaki M, Matsumoto M, Nakamura M, Okawa A, Kawaguchi Y (2016). Prevalence and distribution of ossified lesions in the whole spine of patients with cervical ossification of the posterior longitudinal ligament a multicenter study (JOSL CT study). PLoS One.

[7] Hicks GE, Fritz JM, Delitto A, McGill SM (2005). Preliminary development of a clinical prediction rule for determining which patients with low back pain will respond to a stabilization exercise program. Arch Phys Med Rehabil.

[8] Fujimori T, Watabe T, Iwamoto Y, Hamada S, Iwasaki M, Oda T (2016). Prevalence, concomitance, and distribution of ossification of the spinal ligaments: results of whole spine CT scans in 1500 Japanese patients. Spine.

[9] Park JY, Chin DK, Kim KS, Cho YE. Thoracic ligament ossification in patients with cervical ossification of the posterior longitudinal ligaments: tandem ossification in the cervical and thoracic spine. Spine (Phila Pa 1976). 2008;33(13):E407–10. 10.1097/BRS.0b013e318175c276 18520926

[10] Yamada T, Yoshii T, Yamamoto N, Hirai T, Inose H, Kato T, Kawabata S, Okawa A. Clinical outcomes of cervical spinal surgery for cervical Myelopathic patients with coexisting lumbar Spinal Canal stenosis (tandem spinal stenosis) a retrospective analysis of 297 cases. Spine (Phila Pa 1976). 2018;43(4):E234–41. 10.1097/BRS.0000000000002289 28614282

[11] Ohtsuka K, Terayama K, Yanagihara M (1986). An epidemiological survey on ossification of ligaments in the cervical and thoracic spine in individuals over 50 years of age. Nihon Seikeigeka Gakkai Zasshi.

[12] Tsuyama N, Kurokawa T (1977). Ossifi cation of the posterior longitudinal ligament in the thoracic and lumbar spine. Statistical report of ossification of the posterior longitudinal ligament for all of Japan (in Japanese). Rinsho Seikei Geka.

[13] Akiyama N, Onari K, Kitao S (1981). Ossifi cation of the posterior longitudinal ligament of the thoracic spine; radiological study (in Japanese). Seikeigeka.

[14] Schuenke M, Schulte E, Schumacher U (2010). THIEME atlas of anatomy general anatomy and musculoskeletal system.

[15] Epstein NE, Schwall G (1994). Thoracic spinal stenosis: diagnostic and treatment challenges. J Spinal Disord.

[16] Hou X, Sun C, Liu X, Liu Z, Qi Q, Guo Z, Li W, Zeng Y, Chen Z (2016). Clinical features of thoracic spinal stenosis-associated myelopathy: a retrospective analysis of 427 cases. Clin Spine Surg.

[17] Miyazawa N, Akiyama I (2007). Ossification of the ligamentum flavum of the cervical spine. J Neurosurg Sci.

[18] Guo Q, Ni B, Yang J, Zhu Z (2011). Simultaneous ossification of the posterior longitudinal ligament and ossification of the ligamentum flavum causing upper thoracic myelopathy in DISH: case report and literature review. Eur Spine J.

[19] Mori K, Yoshii T, Hirai T, Iwanami A, Takeuchi K, Yamada T, Seki S, Tsuji T, Fujiyoshi K, Furukawa M, Nishimura S, Wada K, Koda M, Furuya T, Matsuyama Y, Hasegawa T, Takeshita K, Kimura A, Abematsu M, Haro H, Ohba T, Watanabe M, Katoh H, Watanabe K, Ozawa H, Kanno H, Imagama S, Ito Z, Fujibayashi S, Yamazaki M, Matsumoto M, Nakamura M, Okawa A, Kawaguchi Y (2016). Prevalence and distribution of ossification of the supra/interspinous ligaments in symptomatic patients with cervical ossification of the posterior longitudinal ligament of the spine: a CT-based multicenter cross-sectional study. BMC Musculoskelet Disord.

